# Artificial intelligence-based miRNA analysis for precision oncology: diagnostic and prognostic insights

**DOI:** 10.3389/fmolb.2026.1749586

**Published:** 2026-03-05

**Authors:** Tauqeer Zehra, Maryam Koopaie, Nishat Fatima, Gowhar Rashid, Iquebal Hasan, Zainab Siddiqui

**Affiliations:** 1 Department of Biotechnology, Era University, Lucknow, India; 2 Department of Oral Medicine, Tehran University of Medical Sciences, Tehran, Iran; 3 Department of Clinical Biochemistry, Sher-I-Kashmir Institute of Medical Sciences, Srinagar, India; 4 Tanner College of Dental Medicine, University of Pikeville, Pikeville, KY, United States; 5 Center for Disease Mapping and Therapeutic Research, Era University, Lucknow, India

**Keywords:** artificial intelligence (AI), biomarkers, cancer diagnosis, deep learning (DL), machine learning (ML), miRNA, precision oncology, random forest (RF)

## Abstract

**Background:**

MicroRNAs (miRNAs), small molecules that fine-tune gene activity, are consistently disrupted in cancer. Found stably in blood and other fluids, their unique cancer-associated patterns offer a promising route for non-invasive detection and monitoring. Merging artificial intelligence (AI) with miRNA analysis could revolutionize our understanding and treatment of cancer; however, reliably integrating these tools into clinics remains challenging.

**Methods:**

A multi-database search was executed until July 2025 using integrated miRNA-related descriptors and AI/ML ontologies such as support vector machine (SVM), random forest (RF), artificial neural network (ANN), logistic regression (LR), principal component analysis (PCA), and hierarchical clustering (HC), to interpret complex miRNA data in cancer. Our focus was on considering research article related to early cancer detection, prediction of patient outcomes, and guiding personalized treatments**.**

**Findings:**

AI models analysing miRNA signatures demonstrate remarkable accuracy [area under the curve (AUC) often exceeding 0.90] in diagnosing various cancers, such as gastric, breast, and lung cancer (LC). For example, SVM proved highly effective for breast cancer (BC) detection. Crucially, AI helps identify small miRNA sets linked to cancer progression, such as a 3-miRNA combination (hsa-let-7i-3p, miR-362-3p, and miR-3651) that predicts disease stage across eight cancers. RF models achieved near-perfect AUCs (1.00) in some validation studies. AI also identifies miRNAs, such as a specific 5-miRNA group in BC, that signal resistance to chemotherapy. However, significant roadblocks persist: fragmented and non-standardized data, AI tools that exhibit disparate performance across demographic groups (evidenced by racial bias in mammography algorithms), and unaddressed validation gaps.

**Interpretation:**

The powerful combination of AI and miRNA biology is reshaping oncology. It enables earlier cancer detection, more accurate forecasts of disease course, and therapies tailored to the individual. Realizing this potential demands AI models that clinicians can understand and trust, diverse datasets to ensure tools work fairly for all patients, and close teamwork across disciplines to integrate these advances into real-world care. This convergence marks a pivotal shift towards proactive, precise, and accessible cancer management globally.

## Introduction

Cancer corresponds to a heterogeneous, malignant condition, characterized by abnormalities in morphology and the dysregulation of both coding and non-coding RNA (Lange et al., 2021). Globally, there were approximately 10.0 million cancer-related deaths, and 19.3 million new cancer cases in 2020 (Sung et al., 2021). This high incidence and mortality rate indicates that cancer is becoming a leading cause of death worldwide. Moreover, 28.4 million occurrences of cancer are predicted worldwide in 2040, an increase of 47% from 2020 (Sung et al., 2021). This represents a significant increase in new cancer diagnosis with limited treatment options and low percentage of cure rate in most cases. A significant aspect for improving survival is early cancer detection in patients who would respond to effective treatment (Blandin Knight et al., 2017).

MicroRNAs (miRNAs) have the potential to serve as early detection cancer biomarkers due to their unique expression patterns in cancer (Chakrabortty et al., 2023), stability in body fluids (Jang et al., 2021), and ability to be detected non-invasively with high sensitivity and specificity. These features make them promising tools for early detection and monitoring of various cancers, potentially improving prognosis and patient outcomes (Jafari et al., 2023). The identification of recurrent alterations within miRNA-encoding genomic regions in patients with B-cell chronic lymphocytic leukemia provided the first evidence linking miRNAs to cancer pathogenesis (Kminkova et al., 2014; Bayraktar et al., 2024; Maura et al., 2015). Several studies have demonstrated that the dysregulation of miRNA expression plays a critical role in tumor initiation and progression, establishing miRNAs as key regulators in oncogenesis (Chakrabortty et al., 2023; Jang et al., 2021; Jafari et al., 2023; Kminkova et al., 2014; Bayraktar et al., 2024; Maura et al., 2015; Negrini et al., 2007; Pekarek et al., 2023; Aravin and Tuschl, 2005; Gallach et al., 2017). Accurate determination of the molecular drivers of oncogenesis and the early detection of cancer rely on identifying cancer-specific miRNA signatures and monitoring their dynamic effects on gene expression over time (Aravin and Tuschl, 2005; Gallach et al., 2017; Lin and Gregory, 2015; Smolarz et al., 2022). However, distinguishing genuine functional associations from spurious correlations between miRNAs and cancer remains challenging, particularly due to inter-patient variability and heterogeneity in tumor biology (Smolarz et al., 2022). Through the integration and analysis of vast datasets from multiple sources, artificial intelligence (AI) and machine learning (ML) techniques can help overcome these obstacles (Zhang et al., 2023; Jiang et al., 2023). AI is a modern technology that addresses complex healthcare system challenges using mathematically and computationally based algorithmic concepts that are analogous to the capabilities of the human brain (Zhang et al., 2023; Jiang et al., 2023).

## miRNA biogenesis; function, and component

Since the discovery of miRNAs in *Caenorhabditis elegans* nearly 3 decades ago, the miRBase database has expanded significantly and now catalogs miRNAs from 271 organisms, including 1,917 entries for humans (Lee et al., 1993; Kozomara et al., 2019; Zheng et al., 2025). miRNA biogenesis begins with the transcription of a primary miRNA (pri-miRNA) from genomic DNA, primarily by RNA polymerase II, although a small subset is transcribed by RNA polymerase III (Shang et al., 2023; Annese et al., 2020; de Mello et al., 2024). miRNA precursor sequences are predominantly located within the introns or exons or in antisense transcripts (Shang et al., 2023; de Mello et al., 2024). miRNAs may therefore arise intragenically, when processed from introns or exons of host genes, or intergenically, when transcribed from independent genomic loci driven by their own promoters (de Mello et al., 2024). This genomic diversity highlights that miRNAs originate from multiple gene loci and are regulated through various transcriptional contexts. Pri-miRNAs are processed by the Drosha-DGCR8 microprocessor complex to generate precursor miRNAs (pre-miRNAs) (Shang et al., 2023; Annese et al., 2020), which are exported to the cytoplasm by Exportin-5 (XPO5) and further cleaved by Dicer to form mature miRNA duplexes (Shang et al., 2023; Vishnoi and Rani, 2023; Kim et al., 2025). One strand (5p or 3p) is selected based on thermodynamic stability and 5′ nucleotide composition, is loaded into Argonaute (AGO) proteins to form the miRNA-induced silencing complex (miRISC), while the passenger strand is degraded (de Mello et al., 2024; Jouravleva and Zamore, 2025). Once incorporated into the miRISC, the mature miRNA guides the complex to complementary sequences within target transcripts, most commonly located in the 3′ untranslated region (3′ UTR). Binding is primarily mediated by the seed region, a highly conserved stretch of 6–8 nucleotides at positions 2–8 from the miRNA 5′ end (Zheng et al., 2025; Bartel, 2009). Depending on the degree of complementarity between the miRNA and its target, miRNA-mRNA interactions result in translational repression, mRNA deadenylation, and/or mRNA decay (Zheng et al., 2025; de Mello et al., 2024; Vishnoi and Rani, 2023; Kim et al., 2025; Jouravleva and Zamore, 2025; Bartel, 2009). In animals, perfect complementarity is rare; instead, partial pairing, especially within the seed region is sufficient to recruit AGO2 and GW182 proteins, which in turn promote deadenylation and decapping of the target mRNA, ultimately reducing protein output. Through this mechanism, miRNAs fine-tune gene expression rather than acting as binary on/off regulators (Vishnoi and Rani, 2023; Kim et al., 2025; Jouravleva and Zamore, 2025; Bartel, 2009).

**FIGURE 1:**
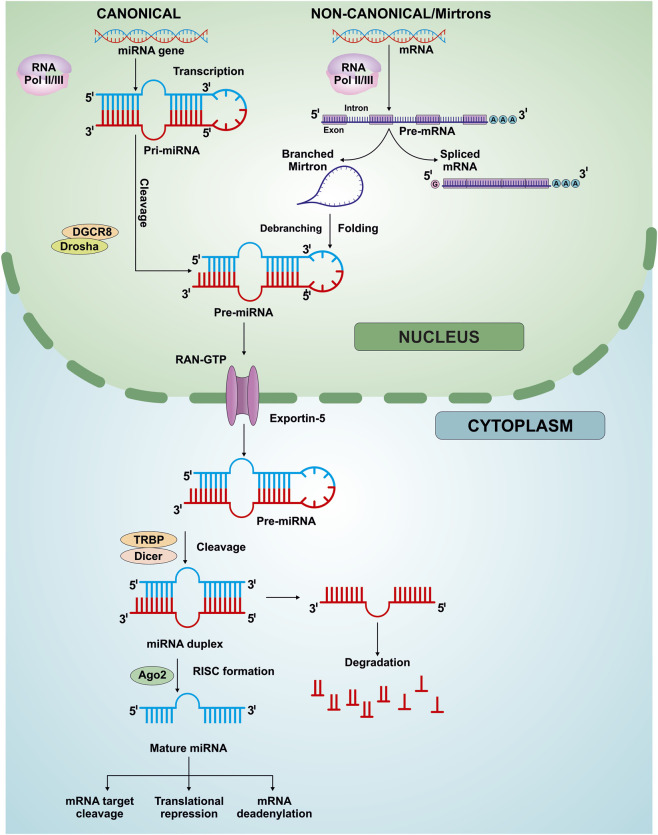
Schematic representation of canonical and non-canonical pathways of miRNA biogenesis and processing. In the canonical pathway, miRNA genes are transcribed mainly by RNA polymerase II, and sometimes by RNA polymerase III to generate primary miRNA transcripts (pri-miRNAs), which are processed in the nucleus by the microprocessor complex comprising Drosha and DGCR8 to generate precursor miRNAs (pre-miRNAs). Pre-miRNAs are exported from the nucleus to the cytoplasm via Exportin-5/Ran-GTP complex, where they are further cleaved by Dicer-TRBP complex to form miRNA duplexes. The guide strand is selectively incorporated into Ago2 containing RISC complex to produce mature miRNA, while the other strand is degraded. In the non-canonical/Mirtrons pathway, pre-miRNA which are the result of mRNA introns or other RNA precursors are not dependent on Drosha-DGCR8 complex. Instead these introns are divided by spliceosome, debranched, and folded into hairpin structures that function as pre-miRNAs. The pre-miRNAs are then exported to the cytoplasm and processed by the Dicer-TRBP complex, thereby bypassing the cytoplasmic processing steps of the canonical pathway. Mature miRNAs regulate gene expression through target mRNA cleavage, translational repression, or mRNA deadenylation.

The biological consequences of miRNA-mediated regulation are extensive, as a single miRNA can target hundreds of transcripts, while individual mRNAs may be regulated by multiple miRNAs (Lee et al., 1993; Kozomara et al., 2019; Bartel, 2009). This many-to-many regulatory architecture enables miRNAs to control entire gene networks and signaling pathways involved in cell proliferation, differentiation, apoptosis, metabolism, and stress responses (Pekarek et al., 2023; Zheng et al., 2025; Vishnoi and Rani, 2023). Dysregulation of miRNA expression can therefore disrupt these networks and contribute to oncogenesis, tumor progression, metastasis, and therapy resistance (Zheng et al., 2025; Annese et al., 2020; Vishnoi and Rani, 2023). These sequential nuclear and cytoplasmic steps of miRNA biogenesis, strand selection, RISC assembly, and downstream gene-silencing mechanism are schematically illustrated in Figure 1.

Epigenetic and post-transcriptional mechanisms, including DNA methylation, histone modifications, and modulation of miRNA biogenesis, are key regulators of miRNA expression (Wang et al., 2018; Ha and Kim, 2014; Leonov et al., 2015; Hill and Tran, 2021; Anand et al., 2010). In epithelial ovarian cancer, miR-98-5p has been shown to target DICER1, leading to reduced DICER1 levels and consequentially altering the expression of downstream miRNAs, such as miR-152 (Wang et al., 2018; Ha and Kim, 2014). This finding illustrates an indirect regulatory layer whereby miRNAs can modulate the miRNA biogenesis machinery itself. Further studies have explored interactions between AGO2, a core component of the RISC, and specific miRNAs. For example, phorbol myristate acetate-induced overexpression of hsa-miR-132 was found to repress AGO2 protein levels while paradoxically increasing AGO2 mRNA expression (Leonov et al., 2015). Reduced AGO2 abundance corresponded with a decreased mature-to-pre-miRNA ratio for miR-221 and miR-146a, indicating impaired miRNA maturation, although AGO2-independent regulatory mechanisms may also contribute to these expression changes (Leonov et al., 2015; Hill and Tran, 2021). These regulatory interactions ultimately affect the maturation and abundance of downstream miRNAs, which function within interconnected networks that can influence cancer-related processes, including angiogenesis and inflammation (Anand et al., 2010).

## miRNA in cancer diagnosis

The seminal discovery linking miRNAs to cancer was reported in 2002 (Orellana and Kasinski, 2015). This pivotal finding catalyzed extensive research documenting dysregulated miRNA expression across diverse cancer types (Orellana and Kasinski, 2015). Aberrant miRNA expression in malignancies arises primarily from chromosomal-abnormalities, including epigenetic modifications (e.g., DNA hypermethylation, hypomethylation, histone remodelling) and genomic alterations (e.g., deletions, amplifications) within miRNA-encoding genomic loci. These aberrations frequently lead to abnormal miRNA gene copy number, leading directly to dysregulated miRNA expression (Zare et al., 2018; Croce, 2009). MiRNAs can function as oncogenes (oncomiRs) or tumor suppressors (tumor-suppressor miRNAs), depending on their specific mRNA targets and expression levels within a given cellular context (Otmani and Lewalle, 2021; Otmani et al., 2022). For example, miR-150 is frequently overexpressed in gastrointestinal cancers. This overexpression enables miR-150 to bind the 3′ UTR of mRNA encoding the tumor suppressor Early Growth Response 2 (EGR2). By repressing EGR2 gene expression, miR-150 promotes carcinogenesis and cellular proliferation (Wu et al., 2010). Conversely, the tumor-suppressor miRNA let-7 plays a critical role in lung cancer (LC) by targeting and suppressing key oncogenes, such as rat sarcoma (RAS) viral oncogene homolog (Yang et al., 2023). Moreover, dysregulation of core miRNA biogenesis machinery components, such as Drosha and Dicer, can also promote tumor development (Ding and Wang, 2025; Zarlashat et al., 2025). Furthermore, diminished Dicer expression is associated with poor clinical outcomes in various cancers such as LC (Szczyrek et al., 2021; Karube et al., 2005) and breast cancer (BC) (Khoshnaw et al., 2012; Caffrey et al., 2013). Critically, despite their frequent dysregulation within tumors, miRNAs remain remarkably stable and readily detectable in patients’ blood samples (Iorio and Croce, 2012).

## Methodology and literature search strategy

A comprehensive literature search was performed to identify studies applying AI and ML techniques to miRNA analysis in precision oncology. Searches were conducted in PubMed, Scopus, Web of Science, IEEE Xplore and Google Scholar from May 2024 to January 2026. In PubMed, both MeSH terms and free-text keywords were used, including: “miRNAs” OR “microRNA” OR “miRNA” OR “miR-,” combined with “AI” OR “ML” OR “deep learning (DL)” OR “neural network” OR “support vector machine (SVM),” using Boolean operators to refine the search. Search strategies were adapted for each database. Eligible studies included original research and reviews applying AI/ML for miRNA-based biomarker discovery, diagnostic classification, prognostic modelling, therapy response, or feature selection in oncology. Irrelevant articles lacking AI methodology or miRNA analyses were excluded. Redundant records were identified and excluded prior to screening. The remaining records were screened based on title, abstract, and full text. Key information regarding datasets, AI models, feature selection methods, validation strategies, and diagnostic or prognostic performance was extracted. Reference lists of included articles were manually explored to identify additional relevant studies. Evidence was synthesized narratively due to heterogeneity in cancer types, datasets, and model architectures.

## Integration of AI and miRNA analysis

The convergence of AI and miRNA research represents a promising Frontier for advancing cancer diagnostics and prognostics. AI enables the analysis of vast, complex biological datasets that exceed human cognitive capacity, facilitating discoveries in miRNA biology (Caudai et al., 2021). ML, particularly DL, facilitates the development of prognostic models and the identification of cancer-associated miRNAs (Aswathy et al., 2024). Circulating miRNAs are inherently stable; tissue specific and reproducibly detectable in bodily fluids making them powerful biomarkers for liquid biopsy-based precision oncology (Gayosso-Gómez and Ortiz-Quintero, 2021; Naranbat et al., 2025). Liquid biopsy enables minimally invasive, longitudinal monitoring of tumor dynamics, supporting early cancer detection, assessment of treatment response, surveillance of minimal residual disease, and early relapse prediction (Aredo et al., 2025; Takizawa et al., 2022). AI provides a robust computational framework for the analysis of high-dimensional circulating miRNA expression datasets derived from liquid biopsies, enabling the extraction of clinically relevant diagnostic and prognostic signatures beyond the capabilities of conventional statistical methodologies (Ling et al., 2022; Shi et al., 2024). The integration of ML algorithms with circulating miRNA profiles supports data-driven therapeutic stratification and dynamic treatment optimization based on the evolving molecular characteristics of individual tumors. Furthermore, predictive modeling approaches facilitate pre-treatment risk classification, identifying patients most likely to respond to standard neoadjuvant regimens while guiding alternative therapeutic strategies for non- responders, thereby strengthening precision oncology paradigms and improving outcome prediction (Aswathy et al., 2024; Christou et al., 2022).

## Overview of key ML algorithms in miRNA analysis

ML approaches applied to miRNA-based cancer research can be broadly divided into supervised or unsupervised learning.

Supervised learning utilizes labeled training data to learn relationships between inputs and outputs, enabling prediction on new samples. Common supervised algorithms include SVM, LR, RF, k-nearest neighbor (KNN), and Naïve Bayes (NB). Elkorany et al. evaluated these algorithms using the Wisconsin BC Diagnostic dataset (WBCD) (Elkorany et al., 2022). In their study KNN achieved the highest accuracy, while NB and LR also demonstrated strong performance. SVM is widely recognized as a highly effective and accurate algorithm for BC diagnosis and prognosis, as supported by multiple studies (Ling et al., 2022; Sarkar et al., 2020; Pal et al., 2016; Yerukala Sathipati and Ho, 2018). Wu et al. evaluated ten common ML algorithms for BC prognosis, among which, multivariate adaptive regression splines (MARS) achieved the highest AUC (Wu et al., 2023).

Unsupervised learning identifies patterns or structures within unlabelled datasets (Ling et al., 2022), and is particularly useful for clustering miRNA expression profiles. HC and PCA are commonly employed to explore underlying patterns and reduce data dimensionality (Stegmayer et al., 2019). For example, applying PCA-based clustering to The Cancer Genome Atlas (TCGA) glioblastoma (GBM) data, Marziali et al. identified a signature of three miRNAs (miR‐23a, miR‐27a, miR‐9‐3p) distinguishing perineural and mesenchymal subtypes (Marziali et al., 2017). ML models leveraging miRNA expression demonstrate high diagnostic accuracy across diverse cancers (e.g., Gastric: 94%, Colorectal: 100%, Breast: 97%, Melanoma: 100%) and non-cancerous conditions (e.g., Gestational Diabetes: 86%, Ischemic Stroke: 96%, Tuberculosis: 83%) (Christou et al., 2022), these studies highlight the robust potential of ML algorithms to analyse complex miRNA datasets for early detection, prognosis and patient stratification.

To better understand these algorithms, we next describe several ML classifiers commonly applied in miRNA analysis and cancer research.

## Support Vector Machine

**FIGURE 2:**
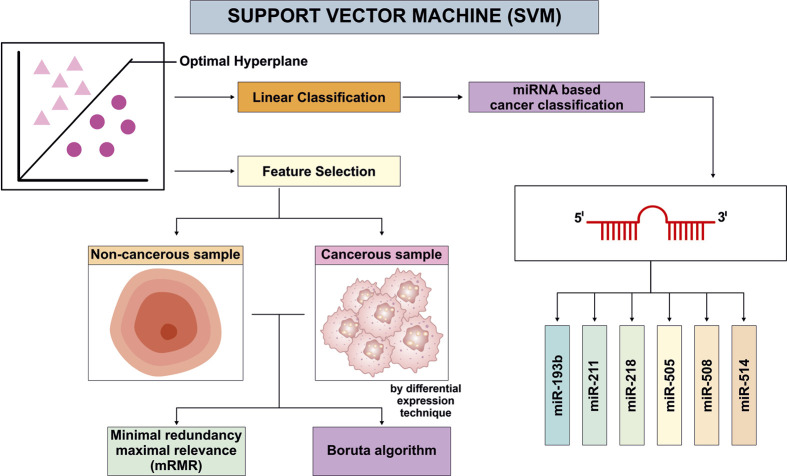
Representative architectures of Support Vector Machine based framework for cancer detection using selected miRNA expression profiles from Dong and Xu (2019).

SVM is a highly effective supervised ML classifier widely used in biological applications, and in miRNA-based cancer biomarker studies as it effectively separates high-dimensional expression profiles into clinically significant categories by identifying an optimal hyperplane in feature space (Salim et al., 2017). In practice, researchers first apply feature-selection (differential expression, Boruta, mRMR, etc.) to reduce the miRNA set, then train via SVM and evaluate performance with cross-validation and ROC/AUC metrics (Salim et al., 2017). For instance, Azari et al. used SVM alongside RF, and KNN on TCGA gastric cancer (GC) data and observed a high AUC for a 29-miRNA panel (with a core panel of hsa-miR-21, hsa-miR-133a, hsa-miR-146b, and hsa-miR-29c focusing on diagnosis and prognosis (Azari et al., 2023). However, SVM models, especially in their standard form, can become biased or unstable when the number of features (for e.g., miRNAs expression) far exceeds the sample size. In this study the Azari et al. started with ∼1882 miRNAs, and even after normalization used 220 miRNAs for ML modelling. Such a high-dimensional feature space relative to a limited sample size increases the risk of overfitting and may compromise the generalizability of the predictive model. Similarly, Dong and Xu developed a 19-miRNA panel (miR-193b, miR-211, miR-218, miR-505, miR-508, and miR-514) by using a SVM to predict survival outcomes and subsequently validate the classification accuracy in ovarian cancer patients. Although the researchers reported the limitation they face in their work, first, the interacting gene and the predictive miRNA did not have experimental validation and second, the sample size with available recurrence information was small (Dong and Xu, 2019). Using SVM for feature selection and classification Sathipati et al., developed CancerSig, a computational approach utilizing a radial basis kernel and the LibSVM library to differentiate early stage from advanced stage cancer based on miRNA expression profiles. Optimizing SVM parameters is crucial for identifying the miRNA profiles. This Pan-cancer analysis identified a panel of three miRNAs (hsa-let-7i-3p, hsa-miR-362-3p, and hsa-miR-3651), significantly contributing a stage prediction across eight cancer types (Yerukala Sathipati et al., 2023). SVM is also used to identify the set of dysfunctional miRNAs, demonstrating ML’s efficacy as biomarker screening tool for cancers (i.e., GC) (Azari et al., 2023; Koopaie et al., 2022). Overall, SVMs are effective for miRNA-based classification, but their results must be interpreted with consideration of certain limitations (Figure 2).

## Random Forest

**FIGURE 3:**
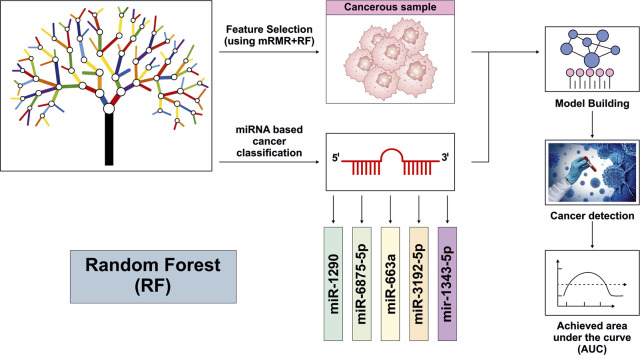
Overview of a Random Forest based framework for cancer detection using miRNA expression profiles from Huang et al. (2022) (Hamidi et al., 2021).

RF is a supervised ML based classifier, originally developed by Breiman, that has become an integral analytical model in miRNA-based oncology due to its ability to model high-dimensional, non-linear regulatory interactions without assuming any distributive hypothesis (Breiman, 2001). The algorithm operates by constructing multiple DTs and creates a forest (Jardillier et al., 2022). DT models applied to miRNA expression profiles identify key miRNAs that hierarchically distinguish cancer patients, enabling transparent and interpretable insight into miRNA-driven disease progression (Hamidi et al., 2021; Huang et al., 2022). A study demonstrated that random survival forest can predict multiple cancer survival developments, but the prognostic signal stability is dependent on sequencing depth (miRNA seq and mRNA seq). Inadequate depth results in reduced model discrimination, suggesting that RF performance is experimentally sensitive to provide information density and unable to completely accommodate for under sampled transcriptomic landscapes (Jardillier et al., 2022). Huang et al. used minimal-redundancy-maximal-relevance (mRMR) feature selection strategy combined with RF to identify five miRNA panel (miR-1290, miR-663a, miR-3192-5p, miR-1343-3p, and miR-6875- 5p). These miRNAs were able to distinguish LC from control samples with an AUC of 0.996 and Matthews Correlation coefficient of 0.9888 (Huang et al., 2022). Despite these results, the RF model provided limited biological interpretability of how each miRNA mechanistically contributes to LC pathogenesis (Huang et al., 2022). Similarly Hamidi et al. applied RF and other classifiers (LR, ANN, XGBoost and DTs) for ovarian cancer prediction, and identified 10 miRNA panel (hsa-miR-5100, hsa-miR-6800-5p, hsa-miR-1233-5p, hsa-miR-4532, hsa-miR-4783-3p, hsa-miR-4787-3p, hsa-miR-1228-5p, hsa-miR-1290, hsa-miR-3184-5p, and hsa-miR-320b) (Hamidi et al., 2021). However, the study had few limitations such as small sample size, no consideration of clinicopathological features and also lacked external validation of the proposed model. In another study, RF model was employed to diagnose BC based on dysregulated miRNA expression profiles, with hsa-miR-139 and hsa-miR-183 identified as key miRNAs contributing to model construction and classification performance (Sherafatian, 2018). Overall, RF provides an effective interpretable model for the identification of miRNA biomarkers, but their performance is dependent on factors, such as adequate sample size, models accuracy, and rigorous experimental and external validation. Consideration of clinical variables including age, tumor stage, tumor grade and cancer associated risk factors are essential to ensure model reliability and generalizability (Figure 3).

## Artificial neural network

**FIGURE 4:**
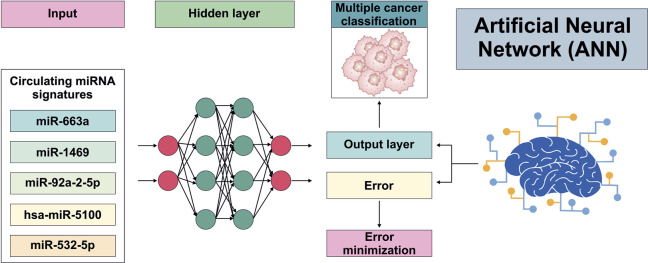
Artificial neural network architecture for multi-cancer detection using circulating miRNA signatures curated from multiple published studies (Huang et al., 2022; Alizadeh Savareh et al., 2020).

ANNs are multilayer, supervised nonlinear ML algorithms, comprising interconnected artificial neurons organized structure into, input, hidden, and output layers (Mohapatra et al., 2018). They process information using a relational method and are capable of modelling high-dimensional dependencies between oncogenic miRNA signatures and tumor development (Sharma et al., 2017). Dynamic adjustment of internal weight structures allows ANNs to capture subtle expression differences, which is helpful in early cancer detection where biomarker detection rates are clinically restricted (Mohapatra et al., 2018). For pancreatic cancer (PC) diagnosis, Saveareh et al. developed an ANN-based classifier utilizing circulating miRNAs (miR-663a, miR-1469, miR-92a-2-5p, miR-125b-1-3p, and miR-532-5p). Their model demonstrated strong diagnostic performance, achieving an accuracy of 0.93 and a specificity of 0.92, and enabled effective patient stratification (Alizadeh Savareh et al., 2020). Similarly, Chi et al. investigated circulating blood miRNAs (hsa-miR-4648, hsa-miR-125b-1-3p, and hsa-miR-3201) for the early detection of PC, reporting diagnostic accuracies exceeding 95%. Meanwhile, the authors acknowledged that they did not employ wet-lab validation (e.g., qPCR) of serum miRNA expression in PC patients. This may limit the robustness of the findings and reduce their translational relevance and real-world clinical applicability (Chi et al., 2023). In ovarian cancer, four miRNAs (hsa-miR-5100, hsa-miR-4532, hsa-miR-4783-3p, and hsa-miR-320b) were identified and demonstrated high stability within an ANN model, achieving an AUC of 1.00 with 100% sensitivity and 100% specificity (Hamidi et al., 2021). Despite these impressive results, the study was based on a small ovarian cancer cohort and a disproportionately large control group, which may limit statistical power and increase the risk of overfitting. Moreover, the GEO dataset used lacked pathological and clinical details such as cancer stage, patient age, and other relevant variables precluding assessment of biomarker performance across clinically meaningful subgroups (Hamidi et al., 2021). Together, present applications of ANN in oncology have expanded, in order to improve diagnostic performance and define molecular information relevance to different miRNAs, however, overfitting due to limited datasets, and lack of external validation remain major challenges. Improved standardization, biological validation, and model interpretability are required for advanced clinical implementation (Figure 4).

## Logistic Regression

**FIGURE 5:**
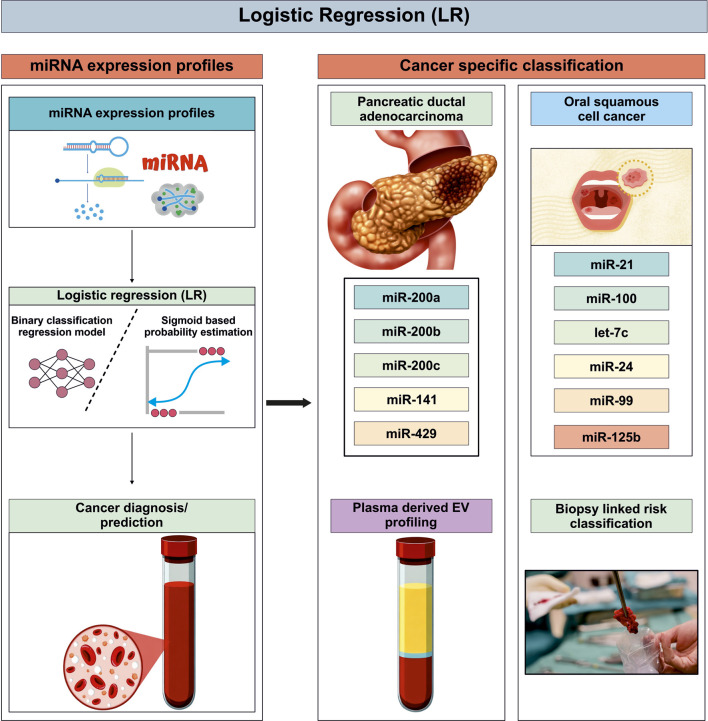
Logistic Regression based cancer classification using miRNA expression profiles curated from multiple published research work for cancer diagnosis and Risk stratification (Pruthi et al., 2024; Liu et al., 2024; Xing et al., 2021).

LR is a regression based statistical and ML method used to model binary outcomes (Pruthi et al., 2024; Liu et al., 2024; Xing et al., 2021). It predicts the probability of an event occurrence by transforming a linear combination of input features into the output probability via a logistic function. LR has been employed to characterize the discriminatory capacity of the miR-200 family (miR-200a, miR-200b, miR-200c, miR-141, and miR-429) for clinical classification in plasma-derived extracellular vesicle profiling studies of pancreatic ductal adenocarcinoma (PDAC). These analyses demonstrated a distinct miRNA expression pattern that differentiates PDAC from benign pancreatic disease (Liu et al., 2024). However, the study was limited by the underrepresentation of patients with advanced-stage disease in the discovery and technical validation cohorts, as such patients typically presented with jaundice at diagnosis rather than being preselected for upfront surgical intervention (Liu et al., 2024). Furthermore, the diagnostic performance was evaluated in a restricted patient population, and the absence of multi-centre and multi-ethnic validation cohorts limits the generalizability and broader clinical applicability of these findings (Liu et al., 2024). Pruthi et al. used LR to develop a biopsy linked miRNA associated tool that uses miR-21, miR-100, let-7c, miR-24, miR-99, and miR-125b to improve cancer prediction thresholds in oral squamous cell cancer (OSCC) risk classification (Pruthi et al., 2024). While the model performed well on the test set, training on only 60 samples (30 cases and 30 control) is small for generalizable ML. This raises concern that the model might over-fit to distinctive features of this dataset, i.e., may perform less well on completely independent data. Indeed the researchers themselves note the relatively small dataset as a limitation (Xing et al., 2021). Also the study lacked external validation as they applied the model only to their own oral potentially malignant disorders cohorts (54 samples), but there was no follow up to see which of those lesions actually progressed to OSCC (Pruthi et al., 2024). Alimena et al. reported that serum miRNA profiles, analyzed using LR, are significantly associated with race and ethnicity, achieving an overall AUC of 0.69 and remaining robust after adjusting for age, menopausal status, and most comorbidities. Of 179 miRNAs profiled, 66 showed significant differences by race/ethnicity, including six of eight miRNAs (hsa-miR-150-5p, hsa-miR-200c-3p, hsa-miR-23b-3p, hsa-miR-29a-3p, hsa-miR-320c, hsa-miR-320d, hsa-mir-32–5p and hsa-mir-92a-3p) previously linked to ovarian cancer risk, highlighting LR’s utility in identifying population-specific miRNA variations that are critical to the development of equitable early detection assays (Alimena et al., 2024). However, the findings lack prospective validation, and no screening performance testing was conducted, limiting their current clinical utility (Alimena et al., 2024). Xing et al. developed a predictive classifier to distinguish chemotherapy-resistant from chemotherapy-sensitive BC using a five-miRNA signature (miR-23a-3p, miR-638, miR-200c-3p, miR-214-3p, miR-451a). They applied LR to miRNA expression data, and found the signature to significantly outperform individual miRNA’s expression (higher AUC) (Xing et al., 2021). However, the study was limited by differences in sample collection sites between the training and independent validation cohorts, which may have introduced technical variability and affected model consistency. Additionally, all samples were derived from Chinese patients, potentially limiting the generalizability of the findings to other populations and ethnic groups (Xing et al., 2021). In summary, LR offers transparent coefficient interpretability and strong clinical adaptability across miRNA-based cancer studies. However, its inherent linear decision structure, sensitivity to multicollinearity and noise, and dependence on low-dimensional feature spaces constrain its diagnostic scalability in highly heterogeneous and complex miRNA biomarker landscapes (Figure 5).

## Decision Tree

**FIGURE 6:**
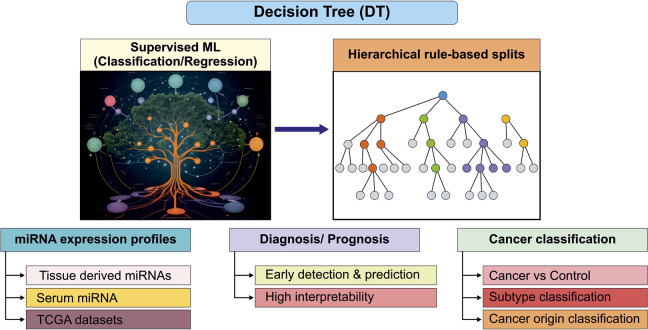
Decision tree model architecture demonstrating hierarchical rule-based splits of miRNA expression data for cancer vs. control and subtype classification.

DTs are simple, interpretable supervised ML techniques used for classification and regression (Costa and Pedreira, 2023; Sherafatian and Arjmand, 2019). They represent decisions as hierarchical tree structures or rule-based splits, enabling clear insight extraction through visual interpretation. DTs effectively identify crucial predictive features with low computational cost (Sherafatian and Arjmand, 2019). DT models have emerged as useful tools in cancer research for analysing miRNA expression profiles to improve diagnostic and prognostic accuracy (Hamidi et al., 2025). Sherafatian and Arjmand applied DT classifiers to LC using miRNA expression data from TCGA and identified hsa-miR-183 and hsa-miR-135b as key discriminative features distinguishing cancerous from normal lung tissue as well as across histological subtypes. When evaluated on independent test data, the models achieved an AUC of 91.2% (Sherafatian and Arjmand, 2019). However DTs split data hierarchically and miss subtle non-linear interactions between miRNA (Sherafatian and Arjmand, 2019). Similarly, Hamidi et al. used ML-based DT and ensemble (collaborative) approaches to serum miRNA profiling in ovarian cancer, identifying a panel of miRNAs including hsa-miR-5100, hsa-miR-6800-5p, hsa-miR-1233-5p, hsa-miR-4532, hsa-miR-4783-3p, hsa-miR-4787-3p, hsa-miR-1228-5p, hsa-miR-1290, hsa-miR-3184-5p, and hsa-miR-320b as potentially informative for early detection and disease prediction (Huang et al., 2022). This suggests that miRNAs may vary according to the type of cancer but are still adaptable to DT based classification frameworks. However, several limitations were noted in the study, including the relatively small ovarian cancer cohort that may cause overfitting. In addition, the absence of key clinicopathological variables such as patient age, tumor stage, and other relevant clinical factors limits the assessment of biomarker robustness and generalizability across diverse patient populations (Huang et al., 2022). Rosenwald et al. validate a qRT-PCR miRNA assays across multiple cancer types and identified hsa-miR-200c and hsa-miR-148b for accurate tissue-of-origin prediction (Rosenwald et al., 2010). Accurate identification of a tumor’s tissue of origin is critical for clinical management. Using miRNA profiling of FFPE samples, a standardized qRT–PCR assay measuring 48 tissue-specific miRNAs was developed, combining a biologically informed binary DT with a KNN classifier. Trained on 356 samples and validated on 204 independent, blinded samples including primary and metastatic tumors the test correctly identified the reference tissue of origin in 85% of cases, with 90% sensitivity for consensus single-tissue predictions (Rosenwald et al., 2010). Overall, DT models are valuable for exploratory analyses, biomarker selection and the development of predictable miRNA diagnostic and prognostic tools, although care must be taken to address overfitting, model generalizability and sample variability (Figure 6).

## Principal component analysis

**FIGURE 7:**
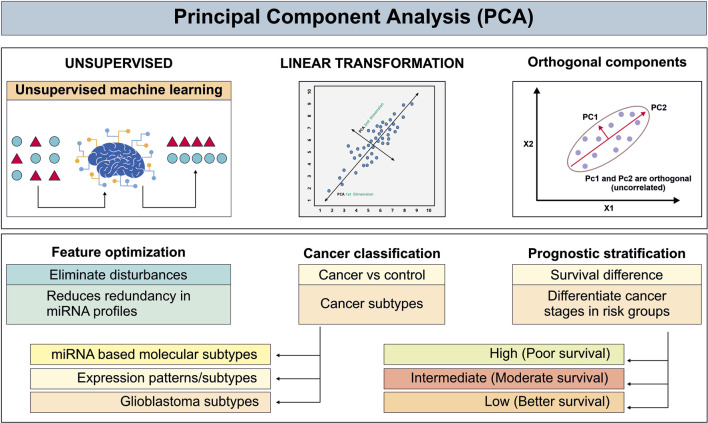
Schematic illustration of principal component analysis for unsupervised miRNA-based cancer classification and prognosis analysis.

PCA, an unsupervised ML dimensionality reduction algorithm, frequently employed in cancer-related miRNA research to extract informative features and eliminate disturbances from high dimensional datasets. It achieves reduction by projecting data onto a new set of orthogonal axes (principal components) capturing maximal variance via linear transformation (Carobene et al., 2022). PCA facilitates more accurate distinction between cancerous and non-cancerous data by applying original variables into orthogonal components, improves model effectiveness, and reduces redundancy in miRNA profiles (Taguchi and Murakami, 2013). Taguchi and Murakami demonstrated that PCA based extraction successfully identified circulating biomarkers including hsa-miR-425, hsa-miR-15b, hsa-miR-185, hsa-miR-92a, hsa-miR-140-3p, hsa-miR-320a, hsa-miR-486-5p, hsa-miR-16, hsa-miR-191, hsa-miR-106b, hsa-miR-19b, and hsa-miR-30d enabling subtype differentiation and diagnostic stratification (Taguchi and Murakami, 2013). However, in this study PCA extracts cancer and non-cancer samples in reduced-dimensional space but it does not reveal which specific miRNAs derive disease pathology, limiting biomarker interpretability. Likewise Marziali et al. applied PCA to GBM miRNA datasets and established a three miRNA prognostic profiles miR‐23a, miR‐27a, and miR‐9, successfully dividing GBM into two clinically distinct subgroups with different survival results (Ling et al., 2022). Although PCA enabled effective subtype stratification, it did not elucidate the mechanistic contributions of the three miRNAs to the observed survival differences, thereby limiting biological interpretability. Moreover, miRNA-mediated signaling in GBM is inherently non-linear, and the linear nature of PCA may therefore overlook subtle regulatory interactions critical for distinguishing prognostic subtypes. In esophageal carcinoma, Gao et al. applied PCA to the miR-144/451 cluster to address multicollinearity. The first two principal components explained 70% of the total variance, sufficiently capturing the cluster’s information (Gao et al., 2016). However this study considered the limitation in complexity of biological regulatory network and the prediction in bioinformatics (Gao et al., 2016). The advantages of PCA in evaluating biological variability have been highlighted by recent multicenter studies. PCA enhanced variance interpretation across multiple clinical sites (Carobene et al., 2022; Taguchi and Murakami, 2013). However, PCA was unable to correct inter-laboratory variations suggesting that pre-analytical standardization remains important (Carobene et al., 2022). Collectively, PCA is useful for miRNA dimensionality reduction, subtype differentiation, and biomarker research across cancer studies. Nevertheless, its reduced interpretability, linearity dependence and sensitivity to pre-processing variations highlight the need for complementary biological validation and advanced or complementary modelling in translational cancer diagnostics (Figure 7).

## Hierarchical clustering

**FIGURE 8:**
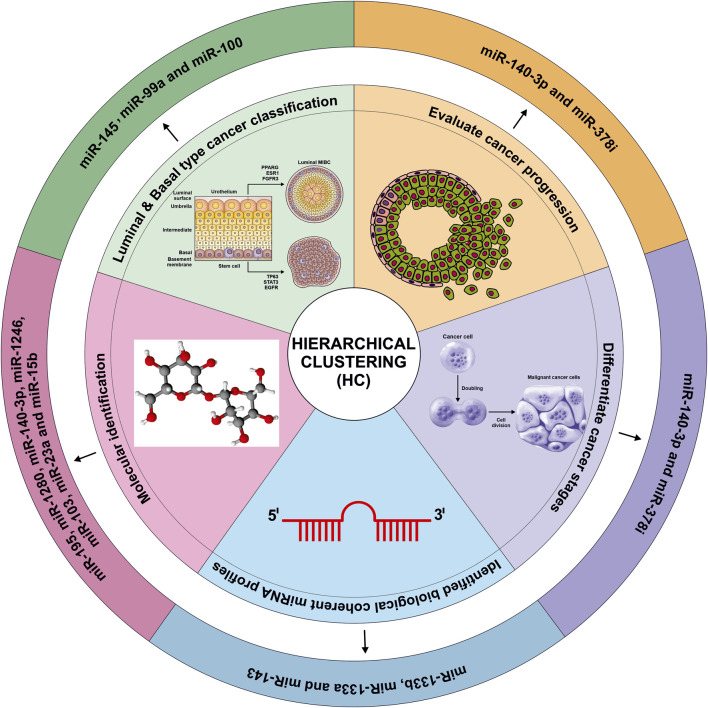
Hierarchical clustering approach for unsupervised analysis of miRNA profiles in cancer classification and prognosis compiled from several published studies (Piepoli et al., 2012; Ochoa et al., 2016; Sugai et al., 2023). GTP, Guanosine Triphosphate; RAN, Ras-related nuclear protein; DGCR8, DiGeorge Syndrome Critical Region Gene 8; TRBP, Transactivation Response Element RNA-Binding Protein; Ago2, Argonaute 2; RISC, RNA-Induced Silencing Complex; RNA pol, PNA polymerase; mRMR, Minimal-redundancy-maximal-relevance; TCGA, The Cancer Genome Atlas.

HC, is an essential unsupervised ML algorithm that group data based on similarity without requiring a predefined number of clusters (Murtagh and Contreras, 2012). HC provides clear dendrograms based visualization of sample relationships, which is particularly useful for exploring heterogeneous miRNA datasets. However, the researchers have observed that HC is highly sensitive to variations and early interactions decision (Murtagh and Contreras, 2012). HC was applied to identify key dysregulated miRNAs such as miR-195, miR-1280, miR-140-3p and miR-1246 in colorectal cancer (CRC), and miR-103, miR-23a and miR-15b in PC, allowing cancer types to be distinguished based on molecular identification (Piepoli et al., 2012). However, the HC becomes insufficient with large feature sets, and this study included hundreds of miRNAs, making the computation heavier and potentially unstable (Piepoli et al., 2012). Similarly Ochoa et al. applied HC to differentiate basal and luminal subtypes of muscle invasive bladder cancer using 15 miRNA panel including miR-133b, miR-133a, miR-143, miR-145, miR-99a, and miR-100. The miRNA profile aligned basal cancer with TCGA cluster IV and luminal with cluster II (Ochoa et al., 2016). However, HC struggles when samples within a biological group differ widely. In this study variations are notable within basal and luminal cancer that could weaken cluster transparency. HC has also been applied to study cancer progression. Sugai et al. applied HC to analyze CRC, revealing stage-associated transitions in miRNA expression, including miR-140-3p and miR-378i. Their study identified paired and dysregulated miRNAs between cancerous and adenomatous components within the same tumor samples (Sugai et al., 2023). Nevertheless, the findings are constrained by the lack of experimental validation and clinical translation. Assao et al. performed HC using R v3.6 and Bioconductor package complex Heatmap. A miRNA panel including miR-181b, miR-21, miR-31, and miR-345 facilitated discrimination between lower squamous cell carcinoma from actinic cheilitis (Assao et al., 2021). Two main clusters were identified, one characterized by downregulated miRNA expression and another exhibited normal to elevated miRNA (Assao et al., 2021). Beyond miRNA studies, the relevance of HC in oncologic imaging has also demonstrated by Rezaeijo et al. HC of mpMRI features was applied to identified biologically distinct intratumoral subregions in prostate cancer. The resulting clusters informed the planning target volumes of image-guided dose painting (delivering higher radiation doses to specific tumor region based on imaging). Radiation doses of 80 Gy, 85 Gy, and 91 Gy were delivered to low, intermediate and high risk regions respectively. This method improved predicted tumor control in high risk regions while preserving normal tissues integrity. Post treatment mpMRI demonstrated reductions in lesions volume. These findings support the feasibility and efficacy of HC-guided dose escalation (Rezaeijo et al., 2021). Collectively, these studies demonstrate that HC is an effective method for identifying cancer subtypes, tumor progression patterns, and biologically coherent miRNA profiles. However, its performance is strongly dependent on sample size, data quality, biological consistency, and feature selection which must be addressed to ensure reliability and clinically useful clustering (Figure 8).

Table 1 shows a comprehensive analysis of some of ML classifiers applied in cancer based miRNA research.

**TABLE 1:** Tabular representation of supervised and unsupervised ML classifiers applied to miRNA expression data for enhanced cancer detection, treatment response prediction and molecular subtyping.

ML model	Cancer	Sample	Sample size	miRNA	Core application	Limitation	Acc/AUC	Ref.
SVM	BC	Breast tissue	386 (193 early stage + 193 advance stage)	hsa-miR-503, hsa-miR-1307, hsa-miR-212, hsa-miR-592, hsa-mir-30a hsa-miR-10a, hsa-mir-375, hsa-mir-107, hsa-mir-378	Differentiate between early vs. advance stage of BC	1. Risk of overfitting 2. Moderate external validation	0.87	Sarkar et al. (2020), Pal et al. (2016), Yerukala Sathipati and Ho (2018)
	LC	Lung tissue	41 (Tumor + adjacent tissue)	hsa-miR-21-5p, hsa-miR-148a-3p, hsa-let-7g-5p, hsa-miR-101-3p, hsa-miR-103a-3p, hsa-miR-431 hsa-miR-200b hsa-miR-22	Differentiate tumor sample from normal sample	1. Lack of experimental validation 2. Reduce generalizability 3. Risk of overfitting	97.83%	Salim et al. (2017), Pal et al. (2016)
	HCC	Hepatic tissue, Saliva	46 (Tumor + control)	hsa-miR-122-5p, hsa-miR-21-5p, hsa-miR-143-3p, hsa-miR-148a-3p, hsa-miR-101-3p, hsa-miR-550a, hsa-miR-574, hsa-miR-424, hsa-let-7i, hsa-miR-549, hsa-miR-518, hsa-miR-512, hsa-miR-3198, hsa-mir-3198-2, hsa-mir-1246, hsa-miR-1246, hsa-mir-3648-2, miR-483-5p, miR-21, and miR-155	Cancer stage prediction, detection of HCC	1. Lack of experimental validation 2. Risk of overfitting 3. Reduce generalizability	95.0%, 74.3%	Salim et al. (2017), Yerukala Sathipati and Ho (2020), Sayed et al. (2024), Mariam et al. (2022)
	Bladder cancer	Tumor tissue	20 (Tumor + control)	hsa-miR-143-3p, hsa-miR-200c-3p, hsa-miR-182-5p, hsa-miR-146b-5p, hsa-miR-103a-3p, hsa-mir-205-5p	Developed a cancer prediction system (Cancer identification)	1.Lack of experimental validation 2. Risk of overfitting	95.0%	Salim et al. (2017)
	Renal cancer	Kidney tissue	24 tumor	hsa-miR-15a hsa-miR-519e	Differentiate tumor sample from normal sample	1. Lack of experimental validation 2. Poor generalizability	0.83	Pal et al. (2016)
	GC	Gastric tissue	536 (465 Tumor + 72 control), 434 (389 Tumor + 45 control)	hsa-miR-21, hsa-miR-133a, hsa-miR-146b, hsa-miR-29c, hsa-miR-139-5p, hsa-miR-139-3p, hsa-miR-146b-5p, and hsa-miR-181a-3p	Cancer diagnosis and prognosis	1. Moderate external validation 2. Lack of biological interpretability 3. Lack of experimental validation	93%	Azari et al. (2023), Wang et al. (2022)
	Ovarian cancer	Ovarian tissue	415 tumor	miR-193b, miR-211, miR-218, miR-505, miR-508 and miR-514	Prognosis of cancer	1. Lack of experimental validation 2. Uncertain clinical utility	0.941	Dong and Xu (2019)
Random Forest	BC	Breast tissue	1175 (1072 Tumor + 103 control)	hsa-miR-139, hsa-miR-96, hsa-miR-18, hsa-miR-25-3p, hsa-miR-505-5p, hsa-miR-29b-2-5p, hsa-miR-10a-5p	Cancer classification and subtype prediction	1. Risk of overfitting	86%	Sherafatian (2018), Contreras-Rodriguez et al. (2023), Sarkar et al. (2021)
	HNSCC	HNSCC Tissue	497 (453 Tumor + 44 Control)	miR-378c and miR-6510-3p	Characterise miRNA expression across HNSCC cancer and its subtypes	1. Limited interpretability of miRNA	0.80	Nunez Lopez et al. (2018)
	LC	Serum	180 (100 Tumor + 80 Control)	miR-1290, miR-663a, miR-3192-5p, miR-1343-3p, miR-6875-5p	Cancer diagnosis	1. Lack of experimental validation 2. Risk of overfitting	0.996	Huang et al. (2022)
	GC	Saliva	38 (19 early stage Tumor + 19 Control)	miR-223-3p, miR 21-5p	Early and non-invasive diagnosis of GC	1. Risk of overfitting	0.93	Koopaie et al. (2024a)
	ESCC	ESCC tissue	226 (113 Tumor + 113 Control)	hsa-mir-124-3p and hsa-mir-1-3p	Cancer diagnosis and treatment by the identification of apoptosis and cancer pathways	1. Lack of experimental validation	N/A	Yadav and Hasija (2025)
	OSCC	OSCC tissue	(167 Tumor + 45 Control)	hsa-mir-124-3p and hsa-mir-1-3p	Cancer diagnosis and treatment by the identification of apoptosis and cancer pathways	1. Lack of experimental validation	N/A	Yadav and Hasija (2025)
ANN	PC	Serum	(81 Tumor + 70 Control)	miR-663a, miR-1469, miR-92a-2-5p, miR-125b-1-3p and miR-532-5p	Non-invasive blood based (circulating miRNA) based diagnostic model generation	1. High computational cost 2. Risk of overfitting	0.93	Alizadeh Savareh et al. (2020)
	CRC	Serum	571 (50 Tumor + 150 Control + 371 from other digestive tract Tumor)	hsa-miR-6726-5p, the hsa-miR-7111-5p, the hsa-miR-1247-3p, and the hsa-miR-614	Diagnosis of cancer	1. Lack of experimental validation 2. Limited interpretability of miRNA	N/A	Afshar et al. (2019)
	Ovarian cancer	Serum	3171 (360 Tumor & 2811 Control)	hsa-miR-5100, hsa-miR-6800-5p, hsa-miR-1233-5p, hsa-miR-4532, hsa-miR-4783-3p, hsa-miR-4787-3p, hsa-miR-1228-5p, hsa-miR-1290, hsa-miR-3184-5p, and hsa-miR-320b	Cancer prediction	1. Risk of overfitting 2. Pathological information such age, stage or other factors are not available	100%	Hamidi et al. (2021)
Logistic Regression	BC	Breast tissue	190 (20 Tumor, 10 chemo sensitive + 10 chemo resistant + 59 internal validation and 71 independent set)	miR-23a-3p, miR-200c-3p, miR-214-3p, miR-451a and miR-638	Identify the neoadjuvant chemotherapy response in breast cancer	1. Variation of sample collection site 2. Lack of population diversity	<0.05	Xing et al. (2021)
	Ovarian cancer	Serum	3171 (360 Tumor + 2811 Control)	hsa-miR-5100, hsa-miR-6800-5p, hsa-miR-1233-5p, hsa-miR-4532, hsa-miR-4783-3p, hsa-miR-4787-3p, hsa-miR-1228-5p, hsa-miR-1290, hsa-miR-3184-5p, and hsa-miR-320b	Cancer prediction	1. Risk of overfitting 2. Pathological information such age, stage or other factors are not available	100%	Hamidi et al. (2021)
	GC	Saliva	38 (19 early stage Tumor + 19 Control)	miR-223-3p, miR 21-5p	Early and non-invasive diagnosis of GC	1. Risk of overfitting	0.919 (AUC)	Koopaie et al. (2024a)
	PC	Serum	26 Tumor	hsa-miR-1246, hsa-miR-205-5p, and hsa-miR-191-5	Identification of pancreatic cancer	1. Small discovery cohort from a single center 2. Dependence on publicly clinical trial data	91.5%	Stegmayer et al. (2019)
	OSCC	OSCC biopsy Tissue, Saliva	114 (30 Tumor + 30 Control 54 from OPMDs)	miR-100,miR-24, miR-99a and Let-7c	Identification of oral squamous cell carcinoma	1. Misclassification of high risk cases 2. Lack of biological representation	0.894	Pruthi et al. (2024), Koopaie et al. (2024b)
Decision Tree	LUAD and LUSC	Lungs tissue	1068 (499 LUAD + 478 LUSC + 91 Control)	hsa-miR-944, hsa-miR-205, miR-205-3p, miR-205-5p, miR-375 and miR-326	Classifying cancer subtypes (LUAD vs. LUSC)	1. Limited ability to capture complex interaction	0.912	Sherafatian and Arjmand (2019), Javanmardifard et al. (2024)
	Ovarian cancer	Serum	3171 (360 Tumor + 2811 Control)	hsa-miR-5100, hsa-miR-6800-5p, hsa-miR-1233-5p, hsa-miR-4532, hsa-miR-4783-3p, hsa-miR-4787-3p, hsa-miR-1228-5p, hsa-miR-1290, hsa-miR-3184-5p, and hsa-miR-320b	Cancer prediction	1. Risk of overfitting 2. Pathological information such age, stage or other factors are not available	91.30%	Hamidi et al. (2021)
PCA	EAC	Esophagus tissue	120 Tumor	hsa-miR-4732-5p, hsa-miR-451a and hsa-miR-144-5p	Cancer detection at early stage	1. Lack of interpretability	N/A	Gao et al. (2016)
	GC	Saliva	38 (19 early stage Tumor + 19 Control)	miR-223-3p, miR 21-5p	Early and non-invasive diagnosis of GC	1. Risk of overfitting	903 (AUC)	Koopaie et al. (2024a)
	Cervical cancer	Cervical tissue, cervical discharge	72 (40 tissue, 19 discharge HSIL + 4 tissue + 4 discharge AIS + 5 control)	miR-125b-1-3p, miR-487b-3p, and miR-1180-3p	Early detection/diagnosis of cervical precancerous lesions	1. Sensitivity to data scaling and outlier	0.974	Suvanasuthi et al. (2025)
	GBM	GBM tissue	35 Tumor	miR-23a, miR-27a, miR-9 (miR-9-3p), miR-10b-3p, miR-34a-5p, miR-193a-3p	Differentiated non-cancerous brain tissues from GBM samples and identify the subtypes of GBM	1. Linear relationship only	N/A	Ling et al. (2022), Grigore et al. (2024)
HC	Bladder cancer	Bladder tissue	405 Tumor	miR-133b, mir-133a, mir-143, miR-145, miR-99a, and miR-100	Differentiation between the two major molecular subtypes of muscle invasive bladder cancer (basal and luminal)	1. Difficulty in choosing the number of clusters and or interpreting dendrograms	85%	Ochoa et al. (2016)
	CRC	Colorectal tissue	33 Tumor	miR-195, miR-1280, miR-140-3p, miR-1246, miR-140-3p miR-378i, miR-542-5p, miR-28-3p, miR-106a-5p, let-7e-5p, miR-454-3p, miR-203a, miR-190a-5p, miR-383-5p, and miR-519a-3p	Differentiation of cancerous sample vs. non-cancerous and identification of CRC	1. Poor performance with high dimensional data 2. Risk of overfitting 3. Single site sampling of tumor	0.91	Piepoli et al. (2012), Sugai et al. (2023), Silva et al. (2021)

BC, breast cancer; PC, pancreatic cancer; LC, lung cancer; GC, gastric cancer; HCC, hepatocellular carcinoma; HNSCC, Head and neck squamous cell carcinoma; ESCC, Esophageal squamous cell carcinomas; OSCC, oral squamous cell carcinoma; CRC, colorectal cancer; LUAD, lung adenocarcinoma; LUSC, lung squamous cell carcinoma; EAC, Esophageal adenocarcinoma; GBM, Glioblastoma; OPMDs, Oral potentially malignant disorders; HSIL, high-grade squamous intraepithelial lesion; AIS, adenocarcinoma *in situ*; Acc, Accuracy; AUC, area under curve.

### Clinical applications of AI-driven miRNA analysis in precision oncology

#### Early cancer detection, subtyping, and pan-cancer early diagnosis

The non-invasive nature and cancer-specific dysregulation of circulating miRNAs make them ideal candidates for early detection (Metcalf, 2024). AI and ML algorithms excel in identifying subtle, multivariate miRNA signatures that distinguish malignant from benign states with high sensitivity and specificity, often surpassing single-marker approaches (Hsu et al., 2026; Yan et al., 2023). Huang et al. used RF classifier with 5-miRNA panel (miR-1290, miR-663a, miR-3192-5p, miR-1343-3p, miR-6875-5p) to distinguish LC patients from healthy controls, achieving an AUC of 0.996 (Hamidi et al., 2021). In prostate cancer, integrating circulating miRNA profiles (miR-21-5p, miR-141-3p, and miR-221-3p) with RF has likewise improved diagnostic accuracy, supporting the clinical applicability of AI-driven miRNA based liquid biopsy (Singh et al., 2025). Similarly, studies of GC have identified diagnostic signatures (miR-21, miR-133a, miR-146b, and miR-29c) using SVM-based feature selection and classification framework (Azari et al., 2023; Khorsandi et al., 2025). DTs provide transparent and clinically interpretable classification rules, facilitating biological insight and clinical adaptation. Sherafatian et al. used a two-step DT model with miR-183 and miR-135b to distinguish lung adenocarcinoma (LUAD) from squamous cell carcinoma, achieving an (AUC = 91.2%) (Sherafatian and Arjmand, 2019). An integrated *in silico* and experimental study identified miR-205-3p/5p, miR-944, miR-375, and miR-326 as robust discriminators between LUAD and LUSC, demonstrating that simple rule-based classifiers using a minimal miRNA panel (miR-944 and miR-326) can achieve near-perfect subtype classification (AUC = 0.98) (Javanmardifard et al., 2024). Unsupervised methods, such as HC have been instrumental in molecular subtyping, revealing miRNA-based clusters in bladder cancer that correspond to basal and luminal phenotypes (Blanca et al., 2023). An emerging Frontier is the development of single-assay, multi-cancer detection platforms, often referred to as pan-cancer early detection tests (Madar et al., 2025). Recent advances in classifiers, often based on ensemble or DL methods, analyse large miRNA panels to detect cancer signals across multiple cancer types and even predict tissue of origin from a blood sample (Metcalf, 2024; Pasha Syed et al., 2022). This approach, moving beyond organ-specific diagnosis, holds immense promise for population-wide screening (Chang et al., 2025).

### AI and miRNA profiling for prognostic stratification and outcome prediction

Beyond detection, miRNA profiles encode information about tumor aggressiveness, disease progression, and patient outcomes (Lotter et al., 2024). AI models uncover miRNA signatures associated with disease stage, recurrence risk, and overall survival, enabling risk-adapted and personalized clinical management (Garg et al., 2025; Eckardt et al., 2025). SVM models have identified minimalist miRNA signatures with pan-cancer prognostic relevance (Anastasiou et al., 2025; Patel et al., 2025). Sathipati et al. identified a 3-miRNA signature (let-7i-3p, miR-362-3p, miR-3651) predictive of cancer stage across eight malignancies (Yerukala Sathipati et al., 2023; Yerukala Sathipati and Ho, 2020). In hepatocellular carcinoma (HCC), SVM models have identified miRNA sets (miR-550a and miR-574) associated with advanced-stage disease (Bosson-Amedenu et al., 2025). Using a statistical learning framework integrating stochastic covariance evolutionary strategy with Cox proportional hazards regression, Sarkar et al. identified 17 miRNAs linked to key oncogenic regulators, including MYC, VEGFA, and AKT1. The identified miRNAs functioned as putative pan-cancer biomarkers across ten cancer types. The proposed model achieved approximately 96% multi-class classification accuracy (Sarkar et al., 2020).Cox regression, coupled with regularization techniques (LASSO/Ridge), and survival RFs represents standard for modelling time-to-event data (Bosson-Amedenu et al., 2025). Studies have developed prognostic indices based on miRNA expression that independently stratify patients into high- and low-risk groups for cancers such as ovarian (Zhou et al., 2022; Han et al., 2023) and GBM (Fang et al., 2025). These models integrate clinicopathological variables with miRNA data to improve predictive accuracy. Unsupervised learning (PCA) and AI-guided differential expression analysis can reveal miRNA clusters associated with metastatic potential (Bukhari et al., 2024). For example, PCA-derived miRNA components have been linked to aggressive phenotypes in prostate cancer (Martínez-González et al., 2021). In a study 11 cancer types were evaluated, mRNA-seq data provided slightly better survival prediction performance than miRNA-seq data, although the absolute differences were modest (Jardillier et al., 2022). These results have important implications for cost-effective study design and large-scale clinical implementation of transcriptomic prognostic models. In Egyptian patients with HCC, ML models based on circulating miRNAs-including miR-483-5p, miR-21, and miR-155 demonstrated high sensitivity (≈92.99) and specificity (≈97.89) for early disease detection. The performance of the SVM-based model exceeded that of conventional statistical approaches (Sayed et al., 2024). Moreover, prognostic miRNA classifiers can identify patients who may benefit from more intensive surveillance or adjuvant therapy, versus those for whom a watchful waiting approach is appropriate, thereby enabling personalized post-diagnostic care (Gandellini et al., 2021; Zhang et al., 2022).

### AI-driven miRNA analysis for predicting treatment response and guiding therapy

The most direct path to personalized oncology is using AI to interpret miRNA profiles that predict therapeutic efficacy or resistance, thereby guiding first-line treatment selection. LR and SVM models are commonly used for this binary classification task. Xing et al. developed a 5-miRNA signature (miR-23a-3p, miR-638, miR-200c-3p, miR-214-3p, miR-451a) using an LR classifier that effectively predicted neoadjuvant chemotherapy response in BC (Xing et al., 2021). Similarly, a SVM classifier using miRNAs discriminated responders from non-responders in BC treatment (Thomopoulou et al., 2021). A study by Contreras-Rodríguez et al. confirmed that models such as SVM and RF often achieve high predictive performance, with RF outperforming SVM in precision metrics when classifying miRNA biomarkers in TCGA BC dataset (Contreras-Rodriguez et al., 2023). Importantly, systematic ML and experimental studies have demonstrated that specific miRNAs are strongly associated with treatment response phenotypes. For instance, higher expression of miR-34a-5p and lower expression of miR-125b-5p were observed in good responders to neoadjuvant chemotherapy. Conversely, upregulation of miR-210, miR-718, and miR-93-3p was associated with poor response and chemoresistance in BC cohorts (Zhang et al., 2021; Richard et al., 2025). Research is exploring miRNAs that regulate pathways targeted by drugs or modulate the tumor immune microenvironment (Roshani et al., 2024). Network analysis combined with ML can identify miRNA-mRNA interaction hubs associated with drug-sensitivity profiles (Amjad and Maghsoodi, 2025; Mao et al., 2025). In GBM, integrated miRNA-mRNA network analyses have shown that dysregulated miRNAs are associated with remodeling of the tumor microenvironment, including alterations in extracellular matrix organization and immune-related pathways (Grigore et al., 2024). Notably, specific miRNAs such as miR-221 and miR-20a were experimentally associated to therapy (temozolomide, a first-line therapeutic agent in GBM) resistance and poor survival, suggesting that miRNA expression profiles contribute to intrinsic treatment response mechanisms in GBM (Palizkaran Yazdi et al., 2024). AI models can be deployed to analyse dynamic changes in miRNA signatures, providing an early readout of treatment effectiveness or emerging resistance, enabling timely therapy switches (Mao et al., 2025). The goal is to shift from a “one-size-fits-all” treatment paradigm to a strategy in which the therapeutic regimen is selected based on a pre-treatment molecular profile, thereby maximizing efficacy and minimizing unnecessary toxicity (Nafchi et al., 2025; Lakshmi et al., 2025).

### Broader application of AI in oncology

Beyond miRNA-centric applications, AI is broadly transforming oncology across diagnostics, prognostics, and therapeutic development, driven by abundant multidimensional biomedical data, and innovative DL architectures (Vosoughi et al., 2025; Tiwari et al., 2025). Its applications span molecular tumor profiling, cancer detection/classification, drug discovery/repurposing, and outcome prediction (Dlamini et al., 2020). AI is transitioning to clinical use, with Chinese trials deploying AI tools for diabetic retinopathy, esophageal/LC screening, and pathology support. AI is poised to revolutionize cancer care delivery (Alowais et al., 2023). In LC screening, AI enhances early detection by analysing multiparametric imaging data (Zhao et al., 2018). DL models can identify malignant pulmonary nodules in chest CT scans with high accuracy (Ardila et al., 2019). Convolutional neural networks (CNNs) enable automated classification of LC histopathology (Pei et al., 2022). Deep neural networks (DNNs) excel at analysing whole-slide images (WSIs), achieving near-perfect performance in tumor detection (AUC >0.99) and distinguishing subtypes (Ehteshami Bejnordi et al., 2017). For example, DeepPATH (based on Inception-v3) accurately classifies TCGA LC WSIs into LUAD, LUSC, and normal tissue (Coudray et al., 2018). AI addresses critical limitations in pathology: sample scarcity, workflow variability, and diagnostic errors (Ardila et al., 2019). Yu et al. extracted 9,879 quantitative features from TCGA LC WSIs. ML (including SVMs and RFs) identified prognostic features stratifying stage I LUAD and LUSC survival, validated in an independent cohort (Yu et al., 2016). AI also integrates multi-omics data (proteomics, genomics, transcriptomics, metabolomics, etc.) to uncover novel therapeutic targets (You et al., 2022). ML and DL approaches have been applied to genome-wide association studies data, epistasis detection, and functional variant interpretation. Tools such as CADD, DANN, Eigen, ExPecto, and DeepVariant enable prediction of variant pathogenicity, gene expression impact, and accurate variant calling from sequencing data (Caudai et al., 2021). Graph-based neural networks (e.g., DeepWalk, graph autoencoders) can embed nodes, integrate multiomics data, and predict miRNA-disease or drug-target associations (You et al., 2022). Another graph attention based model moGAT has been reported to predict pan-cancer subtype (AUC ≈ 0.92) by explicitly modeling topological dependencies across genomic, epigenomic, and transcriptomic graphs (Hsu et al., 2026). CNN models can also automatically quantify immunohistochemistry staining, such as PD-L1 and HER2, with pathologist-level accuracy (Hsu et al., 2026). In HCC, Zhou et al. developed a 3D based DL model, termed 3D SE-DenseNet. This model integrates squeeze-and-excitation blocks into a DenseNet architecture. It was designed to automatically grade HCC using dynamic contrast-enhanced MRI. The proposed model achieved an accuracy of 83%, outperforming the baseline DenseNet (72%), thereby demonstrating the potential of non-invasive MRI-based DL approaches for HCC grading (Zhou et al., 2019). Nagpal et al. build and validate a DL system to automatically perform Gleason grading on prostate cancer. The model was trained on thousands of digitized slides and validated against expert pathologists. It achieved 71.7% accuracy comparable to experienced pathologists (58.0%), improving reproducibility and consistency in prostate cancer grading (Nagpal et al., 2019). Similarly, Bhinder et al. employed DNN classifier-based model to predict Gleason Scores using WSI for H&E-stained prostatectomy tissues (Bhinder et al., 2021). The group reported an improved prediction accuracy of Gleason Scores estimated from their models (κ = 0.70) compared to those determined by a panel of 29 independent pathologists (κ = 0.61) (Bhinder et al., 2021). AI further enables early detection through minimally invasive liquid biopsies analysing circulating tumor DNA (ctDNA) and cell-free DNA (cfDNA) (Gayosso-Gómez and Ortiz-Quintero, 2021; Naranbat et al., 2025; Aredo et al., 2025; Takizawa et al., 2022; Shi et al., 2024). Chabon et al. developed Lung-CLiP, a ML method predicting ctDNA presence in LC patients’ blood (Chabon et al., 2020). This technique first assesses tumor association probability of cfDNA mutations using fragment size features and a flexible net model. Notably, Mouliere et al. achieved higher accuracy (AUC = 0.91–0.99) for pan-cancer ctDNA detection using a RF classifier trained on cfDNA fragmentation patterns (Hsu et al., 2026).

ML based algorithms like trRosetta and AlphaFold predict accurate 3D protein structures from sequences, facilitating druggability evaluation. GraphDTA represents drugs as molecular graphs and proteins as sequences to predict continuous binding affinities. AI can also be used to optimize CRISPR-Cas9 guide RNA (gRNA) design by predicting on-target efficiency and minimizing off-target effects. Notable tools include CRISTA, DeepCRISPR, CROTON, CRISPR-ONT, and CRISPR-OFFT**.** Integration of CRISPR perturbation with single-cell RNA sequencing has further enabled large-scale mapping of genetic interactions (Caudai et al., 2021). While network based tool such as PockDrug identified TNIK as a druggable pocket among thyroid cancer targets HEY2, TNIK, and LRP4 (You et al., 2022). A study mentioned the prediction of synthetic lethal interactions, which guide targeted anticancer therapies. Tools such as DiscoverSL, EXP2SL**,** and ensemble ML approaches leverage multiomic cancer data to identify actionable vulnerabilities (Caudai et al., 2021).

AI applications now span medical imaging, automated diagnosis, drug discovery, and personalized treatment. AI-enhanced imaging, pathology, and treatment planning not only reduce clinician workload but also improve diagnostic sensitivity and accuracy (Caudai et al., 2021; Hsu et al., 2026; Yan et al., 2023; You et al., 2022).

### Challenges of AI in oncology

AI demonstrates capabilities in supervised learning, robustness to noisy data, and modelling highly complex nonlinear interactions (He et al., 2019). While AI aids in early cancer detection, potentially reducing morbidity and mortality, its applications in clinical oncology extend far beyond diagnosis (Hu et al., 2025). ML and DL, key subfields of AI, are grounded in statistical modelling and inference. These fields enable outcome and feature prediction by learning iteratively from data (Melarkode et al., 2023). DL has been particularly transformative in medical imaging analysis (Chartrand et al., 2017), while a growing body of research highlights ML’s diagnostic and prognostic accuracy (Ferroni et al., 2019). In specific, well-controlled clinical settings, such systems can outperform radiologists by increasing sensitivity and reducing false positives. However, their performance may degrade outside curated training environments, underscoring the persistent risk of overfitting and limited generalizability (Chen and Asch, 2017). This reflects a fundamental trade-off in ML between complexity and interpretability. Highly complex models (e.g., neural networks and RFs) often achieve higher accuracy but are less interpretable, whereas simpler models (e.g., LR, DTs) offer transparency at the potential cost of performance (Huang et al., 2020). Despite rapid advancements, several significant barriers continue to impede its routine clinical integration in oncology. These include limited availability of high-quality, well-annotated datasets; challenges related to data acquisition, standardization, and secure sharing; and the need to ensure robust patient privacy and data protection. Furthermore, technical limitations, ethical considerations, and regulatory uncertainties remain substantial obstacles. Ensuring consistent and equitable performance of AI systems across diverse patient populations is particularly critical to avoid bias and disparities in care (Lotter et al., 2024; Sebastian and Peter, 2022; Topol, 2019). However, expert-driven data annotation is inherently time-intensive and heavily reliant on scarce clinical expertise, placing a substantial burden on clinicians and domain specialists. This annotation process requires considerable effort and consistency to ensure data quality and validity. Further complicating these efforts is the lack of standardized cancer data representations and the fragmented storage of predominantly unstructured information across heterogeneous electronic health record (EHR) systems, which limits data interoperability, scalability, and downstream model generalizability (Sebastian and Peter, 2022).

Data security and privacy preservation remain major concerns in the clinical deployment of AI systems (Topol, 2019). Despite substantial enthusiasm surrounding “big data” and ML, relatively few AI tools have successfully transitioned into routine clinical practice (Hosny et al., 2018; Handelman et al., 2018). Obermeyer et al. emphasize the importance of complementing ML approaches with robust statistical frameworks to ensure methodological rigor and clinical relevance in medicine (Obermeyer and Emanuel, 2016). Framing AI as “smarter” than clinicians is ultimately unproductive; instead, its true value lies in augmenting human decision-making through the integration of large-scale, multimodal clinical data (Chen and Asch, 2017).

Ethical implementation of AI in oncology is inherently complex and highly context-dependent. The accuracy and reliability of AI systems are critically contingent on the quality, representativeness, and curation of training data, as well as algorithmic design choices. Because AI models generate probabilistic predictions, errors are unavoidable in certain clinical scenarios. However, legal and regulatory frameworks that clearly define accountability and liability for AI-related errors or patient harm remain insufficiently developed.

AI-based oncology tools hold considerable promise for resource-limited settings where access to specialized expertise is constrained. Nevertheless, reliance on proprietary or third-party AI models complicates transparency, bias detection, and mitigation strategies. Moreover, the development of high-performing models often requires extremely large datasets, which may exacerbate patient concerns and anxiety when data usage and governance practices lack transparency (Sebastian and Peter, 2022). Ensuring equitable AI performance across diverse populations is therefore essential (Chen et al., 2023).

Paradoxically, AI also offers significant opportunities to reduce healthcare disparities (Badal et al., 2023). This duality is particularly pronounced in oncology, where bias can be introduced at every stage of the AI lifecycle from data collection and model development to clinical deployment and patient interaction. BC screening has been extensively studied as a paradigmatic use case for evaluating AI-driven equity in healthcare. Persistent disparities in access, screening utilization, and outcomes among Black women and other underserved populations are well documented (Srivastav et al., 2025). AI systems have the potential to either reinforce or mitigate these inequities. For example, mammography algorithms trained predominantly on data from white patients have demonstrated reduced performance in more diverse populations (Hsu et al., 2022). In contrast, initiatives such as the EMBED project aim to construct inclusive, representative datasets (Jeong et al., 2023), and several recent models have shown robust performance across demographic subgroups (Lotter et al., 2024; Hsu et al., 2022).

The development of robust AI models for miRNA-based oncology is challenged by several critical technical limitations. The high dimensionality of miRNA expression data relative to typically small sample sizes substantially increases the risk of overfitting, whereby models capture noise rather than biologically meaningful patterns and consequently fail to generalize beyond the training cohort. This necessitates rigorous validation strategies, dimensionality reduction, and feature selection approaches to ensure model robustness and reproducibility (Aswathy et al., 2024; Azari et al., 2023; Dong and Xu, 2019; Jardillier et al., 2022; Hamidi et al., 2021; Huang et al., 2022; Xing et al., 2021; Chen and Asch, 2017; Kang and Kim, 2025; Aghayousefi et al., 2023).

In addition, limited and class-imbalanced datasets frequent in oncological studies compromise statistical power and model stability, while predisposing algorithms to favor majority classes. Addressing these issues often requires specialized methods, such as resampling techniques including the Synthetic Minority Over-sampling Technique (SMOTE), as well as the use of evaluation metrics better suited for imbalanced data, such as the area under the precision–recall curve (AUPRC) (Yang and Mirzaei, 2024; Peng et al., 2023). Finally, batch effects arising from technical variability in sample collection, processing, and measurement platforms can obscure true biological signals and severely limit the portability and generalizability of miRNA signatures across studies and clinical settings. Mitigating these effects demands careful experimental design, standardized protocols, and advanced normalization and batch-correction methods (Azari et al., 2023; Dong and Xu, 2019; Jardillier et al., 2022; Hamidi et al., 2021; Huang et al., 2022; Xing et al., 2021; Düren et al., 2022; Ben-Elazar et al., 2021). Collectively, addressing these interconnected challenges is essential for translating miRNA-based AI models into reliable, clinically actionable oncology tools.

## Future direction

Beyond early detection, diagnosis, and treatment, AI’s impact will extend into daily living and long-term survivorship (Lee and Yoon, 2021). However, technological advancement alone will not drive AI’s future in oncology. Its trajectory will be shaped by addressing several critical needs. Stakeholders, including clinicians, researchers, administrators, and IT specialists, must collaboratively evaluate key criteria during AI development and implementation (Lotter et al., 2024). First, unbiased evaluation of AI’s impact using clinically significant, cancer-specific outcome metrics is paramount. This requires increased funding for pragmatic research and randomized trials assessing AI’s generalizability across diverse demographics and clinical settings. Second, the clinical benefits of AI must be rigorously evaluated alongside compatible reimbursement models. Value-based care principles, measuring health outcomes achieved per dollar spent, should guide investment decisions (Elemento et al., 2025). Third, enhancing prognostic models is critical. Current AI-based outcome prediction in oncology (e.g., for oral cancer) is limited by insufficient incorporation of sociodemographic and clinicopathological variables. Future research should focus on (a) Improving AI’s ability to classify cancer stage probability and optimize treatment selection. (b) Leveraging multi-level data to identify causal relationships between variables. (c) Integrating diverse data sources to enhance model interpretability and accuracy. Techniques like interpretable DL and explainable AI are crucial here (Shao et al., 2021). Fourth, bridging disciplinary divides is essential. Historically, perspectives on AI in healthcare have been polarized. Model effectiveness hinges on collaboration between computational experts and biomedical scientists. Initiatives like Moon-shot unite patients, researchers, advocates, and clinicians to accelerate progress. Their goals include fostering collaboration, enhancing cancer data sharing, and accelerating discovery (Sebastian and Peter, 2022). Fifth, technical refinement for specific applications is crucial. For skin cancer detection using dermoscopy, future research should focus on optimizing methods like CNN-based Deep Siamese domain adaptation with the Honey Badger algorithm. Key priorities include diversifying training datasets, incorporating multimodal data (e.g., clinical history), improving model interpretability, advancing transfer/few-shot learning to address data scarcity, enabling real-time mobile deployment, and conducting thorough validation and outcome studies (Qureshi and Roos, 2023). These advances aim to maximize diagnostic accuracy, clinical utility, and accessibility (Hussain and Toscano, 2024). Sixth, advancing personalized oncology. AI can analyse high-volume genomic and molecular data to enable truly individualized treatment regimens. Research should focus on developing AI techniques that determine optimal therapies based on unique patient profiles (Dixit et al., 2023). Seventh, integrating AI with multimodal diagnostics. AI should be combined with traditional methods (e.g., biopsies, lab tests) to maximize diagnostic accuracy. While significant progress exists in AI for diagnosis/prognosis, prior studies confirm ML/DL accuracy for detecting cancers like oral cancer, aiding experts and reducing diagnostic errors. However, DL often surpasses ML in early detection accuracy of oral cancer. Beyond predicting lesion progression, AI offers strategies to complement existing techniques for improved identification of oral cancer and oral potentially malignant disorders. Future research should thus prioritize multimodal data fusion algorithms integrating histology, clinical, imaging, and molecular data for early diagnosis and improved outcome prediction (Dixit et al., 2023).

## Conclusion

The widespread dysregulation of miRNAs across cancer types, coupled with their stability in body fluids and central role in gene regulatory networks, establishes miRNAs as important biomarkers for precision biology. This review demonstrates that AI-based approaches improve conventional statistical and single biomarker method by effectively modelling high-dimensional, non-linear, and heterogeneous miRNA sequences. The identification of compact although highly informative miRNA profiles is made possible by ML algorithms such as SVM, RF, PCA, ANN and collective models. The models consistently demonstrate higher diagnostic, prognostic and predictive performance across a wide range of cancers. For successful translation into clinical practice, various significant approaches must be implemented within translational medicine. These include standardization of miRNA sample processing and analytical methods, conducting thorough external and prospective validation across multi-center and demographically diverse cohorts, and implementing transparent AI frameworks to improve interpretability and clinician confidence. Integrating AI-driven miRNA models with clinicopathological data, imaging outcomes, and complementary omics layers are also essential to improve robustness and real world applicability. AI-driven miRNA sequences provide practical tools for minimally invasive liquid biopsies for molecular subtyping, early detection of cancer, and chronic disease screening in clinical perspective. By identifying patients who are most likely to respond to specific treatments or developing resistance, predictive miRNA-based models can help clinicians in therapeutic diversification. This enables for personalized treatment selection and minimizes unnecessary damage. Researchers should focus on dependable models, standardized reporting of performance metrics, and regulatory aligned data exchange framework.

The combination of AI, ML and miRNA studies offers an effective template for advancing precision cancer research and encouraging the incorporation of data-driven molecular biomarkers into routine cancer treatment.

## Glossary

AGO: Argonaute
AI: Artificial Intelligence
AUC: Area Under the Curve
BC: Breast Cancer
cfDNA: Cell-Free DNA
CNN: Convolutional Neural Network
CRC: Colorectal Cancer
ctDNA: Circulating Tumor DNA
DL: Deep Learning
DNN: Deep Neural Network
DT: Decision Tree
EGR2: Early Growth Response 2
GBM: Glioblastoma
GC: Gastric Cancer
ANN: Artificial Neural Network
HC: Hierarchical Clustering
HCC: Hepatocellular carcinoma
KNN: K-Nearest Neighbor
LR: Logistic Regression
LUAD: Lung Adenocarcinoma
LUSC: Lung Squamous Cell Carcinoma
miRNA: MicroRNA
ML: Machine Learning
mpMRI: Multiparametric Magnetic Resonance Imaging
MRI: Magnetic Resonance Imaging
NB: Naïve Bayes
PCA: Principal Component Analysis
PC: Pancreatic Cancer
PDAC: Pancreatic Ductal Adenocarcinoma
pri-miRNA: Primary MicroRNA
pre-miRNA: Precursor MicroRNA
RAS: Rat Sarcoma
RF: Random Forest
RISC: RNA-Induced Silencing Complex
ROC: Receiver Operating Characteristic
SVM: Support Vector Machine
TCGA: The Cancer Genome Atlas
TRBP: Transactivation Response Element RNA-Binding Protein
UTR: Untranslated Region
LC: Lung Cancer
WSI: Whole-Slide Imaging
XPO5: Exportin 5
GTP: Guanosine Triphosphate
RAN: Ras-related nuclear protein
DGCR8: DiGeorge Syndrome Critical Region Gene 8
RNA pol: RNA polymerase
mRMR: Minimal-redundancy-maximal-relevance
